# Doença de Danon: Compreendendo o Papel das Variantes do *LAMP2* na Miocardiopatia e no Envolvimento Multissistêmico

**DOI:** 10.36660/abc.20250186

**Published:** 2026-04-17

**Authors:** Giovanna Napolitano Pereira Ribeiro, Layara Fernanda Vicente Pereira Lipari, Paula Mendonça Senra, Lucas Vieira Lacerda Pires, Marjorie Hayashida Mizuta, Natália Quintella Sangiorgi Olivetti, Vera Demarchi Aiello, José Eduardo Krieger

**Affiliations:** 1 Hospital das Clínicas da Faculdade de Medicina da Universidade de São Paulo Instituto do Coração Laboratório de genética e cardiologia molecular São Paulo SP Brasil Laboratório de genética e cardiologia molecular - Instituto do Coração do Hospital das Clínicas da Faculdade de Medicina da Universidade de São Paulo, São Paulo, SP – Brasil

**Keywords:** Lisossomos, Cardiomiopatia Hipertrófica, Doenças Genéticas Inatas

## Abstract

A doença de Danon é uma doença rara ligada ao cromossomo X, causada por variantes patogênicas ou provavelmente patogênicas no gene da proteína 2 da membrana associada ao lisossomo (*LAMP2*). Manifesta-se principalmente como cardiomiopatia hipertrófica, miopatia, deficiência intelectual e retinopatia, sendo que os homens geralmente apresentam um fenótipo mais grave e de início precoce. A fisiopatologia da doença de Danon está ligada à autofagia defeituosa devido à deficiência de *LAMP2*, levando ao acúmulo de vacúolos cheios de glicogênio dentro dos cardiomiócitos e outros tecidos. Isso resulta em hipertrofia cardíaca progressiva, anormalidades de condução e eventual insuficiência cardíaca, frequentemente necessitando de transplante cardíaco. O envolvimento da musculatura esquelética é comum em homens, enquanto o comprometimento cognitivo é observado em ambos os sexos, embora mais prevalente em homens. O diagnóstico é desafiador devido à raridade da doença e à apresentação variável, o que destaca a necessidade de uma abordagem multidisciplinar, incluindo testes genéticos para um diagnóstico definitivo. Os testes genéticos devem ser realizados somente após aconselhamento genético adequado. O diagnóstico diferencial deve ser considerado quando há suspeita de doença de Danon, dependendo dos sinais e sintomas, incluindo doenças como a doença de Fabry, a síndrome *PRKAG2*, a doença de Pompe, entre outras. As estratégias de tratamento atuais focam no alívio dos sintomas, sendo o transplante cardíaco uma intervenção crucial para casos avançados. Terapias emergentes, como a terapia gênica direcionada ao gene *LAMP2*, podem auxiliar na alteração da progressão da doença. Esta revisão oferece uma visão abrangente da doença de Danon, abordando suas manifestações clínicas, mecanismos subjacentes, desafios diagnósticos e potenciais abordagens terapêuticas para melhorar os resultados. Nosso objetivo é aumentar o reconhecimento da doença de Danon para que a prevenção de possíveis complicações e o diagnóstico precoce se tornem viáveis, levando, em última análise, a desfechos mais favoráveis para os pacientes.

## Introdução

### Entendendo a Doença de Danon: Genética, Manifestações Clínicas e Fisiopatologia

A doença de Danon (DD) é uma doença dominante ligada ao cromossomo X, causada por variantes germinativas no gene da proteína 2 da membrana associada ao lisossomo (*LAMP2*). Clinicamente, caracteriza-se por cardiomiopatia hipertrófica (CMH), miopatia, deficiência intelectual (DI) e retinopatia.

A condição foi descrita pela primeira vez por Danon et al. em 1981, em um relato de dois meninos com cardiomiopatia, miopatia e DI.^1^ Em 2000, foram relatados casos adicionais, incluindo 10 pacientes não relacionados com miopatia vacuolar e CMH, todos apresentando deficiência na proteína *LAMP2* e variantes no gene *LAMP2.*^2,3^

A DD apresenta um espectro de sintomas e, devido à sua herança dominante ligada ao cromossomo X, tanto homens hemizigotos quanto mulheres heterozigotas podem ser afetados.^4^ Os homens tipicamente desenvolvem hipertrofia ventricular esquerda concêntrica progressiva, frequentemente necessitando de transplante cardíaco.^5^ Anormalidades na condução cardíaca também são comuns. A fraqueza muscular esquelética pode levar a atraso na aquisição de marcos motores, dificuldades de aprendizagem ou DI. Nas mulheres, a doença tende a se manifestar mais tarde e progredir mais lentamente, com fenótipos mais variáveis.^4,6,7^

A DD é um desafio diagnóstico, o que enfatiza a importância de uma abordagem multidisciplinar para um diagnóstico preciso, incluindo aconselhamento genético sensível e estratégias terapêuticas personalizadas. À medida que nossa compreensão da fisiopatologia da DD se aprofunda, há esperança de novos tratamentos e melhores resultados. Compartilhar conhecimento sobre doenças raras como a DD é essencial para ajudar os médicos a suspeitar e diagnosticar corretamente pacientes potencialmente afetados, mitigando assim as complicações.

## Método

Os autores realizaram uma revisão sistemática da literatura seguindo metodologia científica estabelecida para reunir e analisar estudos relevantes sobre a DD no campo da cardiogenética. Uma busca abrangente foi realizada em diversas bases de dados acadêmicas, incluindo PubMed, Embase e Cochrane, utilizando as palavras-chave: "*Danon disease*"; "*cardiomyopathy*"; "*LAMP2*" e "*lysosomal storage disorders*". Os critérios de seleção priorizaram artigos publicados na última década que abordassem diretamente os aspectos genéticos e moleculares da doença. A revisão focou particularmente em artigos que exploraram os mecanismos moleculares envolvidos na fisiopatologia da DD.

Durante o processo de revisão, os autores também encontraram alguns resultados conflitantes entre os estudos selecionados, os quais foram cuidadosamente analisados e discutidos em conjunto, considerando as diferenças metodológicas e as variações nas populações estudadas, nas definições de desfecho e nas abordagens analíticas. Todas as figuras e tabelas presentes neste artigo foram elaboradas pelos autores, com base em dados extraídos de artigos previamente publicados.

### Epidemiologia

#### Prevalência global e tendências de diagnóstico da doença de Danon

A prevalência da DD permanece incerta, mas se sabe que afeta indivíduos em diversos países e etnias. A cardiomiopatia de Danon é progressiva e tipicamente caracterizada por um fenótipo de hipertrofia ventricular esquerda. Entre pacientes pediátricos com CMH, a DD foi identificada como a causa subjacente em 4% dos casos.^5^ Em certos subgrupos, a prevalência é notavelmente maior: até 17% em indivíduos com espessamento da parede ventricular esquerda e pré-excitação^8^ e até 33% em pacientes do sexo masculino que apresentam miopatia vacuolar na biópsia muscular e CMH.^9^ De acordo com um artigo recente, os homens tendem a apresentar CMH em 96,2% dos casos, enquanto as mulheres podem apresentar CMH (70,3%) ou cardiomiopatia dilatada (29,3%). Mas, quando se trata de transplante cardíaco, como a doença continua a progredir, ocorre igualmente em ambos os sexos.^4,10^

### Fisiopatologia

#### Disfunção da *LAMP2* na doença de Danon: Mecanismos e impacto celular

O gene *LAMP2*, localizado no cromossomo Xq24, codifica uma glicoproteína fundamental da membrana lisossomal, crucial para a biogênese do lisossomo, a regulação do pH lisossomal e a autofagia.^11–16^ A *LAMP2* regula o pH do lúmen lisossomal, facilitando a acidificação lisossomal para uma atividade hidrolítica ideal.^16^ Ela também desempenha um papel significativo na autofagia mediada por chaperonas, um processo essencial para a degradação lisossomal de proteínas sob estresse e durante a renovação proteica normal.^12–15^ A glicoproteína *LAMP2* também é necessária para a fusão adequada entre autofagossomos e lisossomos durante a autofagia.^14^

Uma variante germinativa patogênica (PV) ou provavelmente patogênica (LPV) no gene *LAMP2* geralmente resulta na perda ou mau funcionamento da proteína *LAMP2*, levando à fusão deficiente de autofagossomo-lisossomo e à degradação prejudicada do autofagossomo, resultando no acúmulo de vacúolos cheios de glicogênio em cardiomiócitos e outros tecidos (como na Figura Central). Existem três isoformas de *LAMP2* — *LAMP2*A, *LAMP2*B e *LAMP2*C — que diferem em seus domínios transmembrana e citoplasmáticos, mas compartilham domínios luminais idênticos.^14,17–20^ As características clínicas da DD são mediadas principalmente pela perda da isoforma *LAMP2*B, expressa no tecido muscular.^21^

**Figure:**
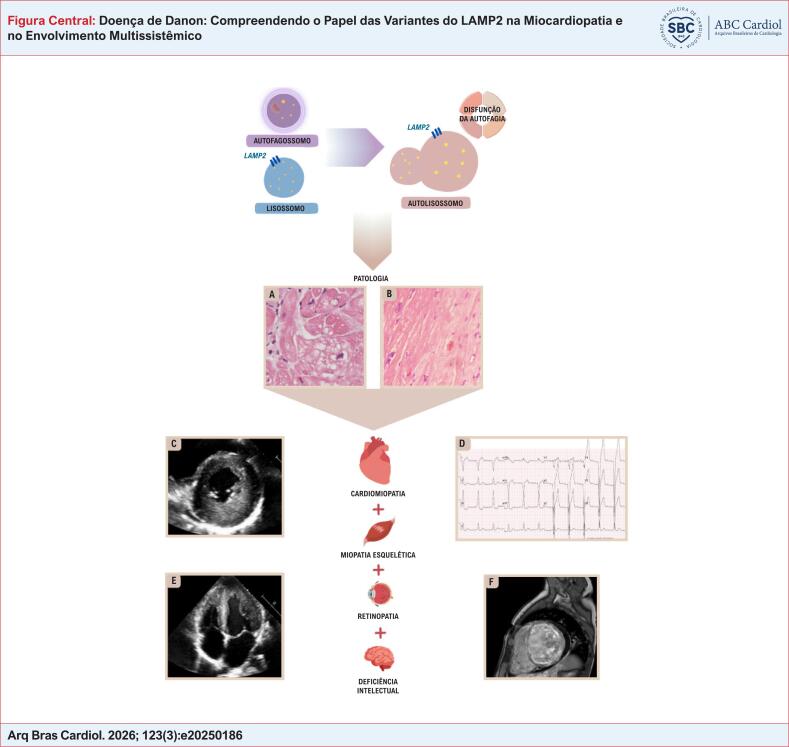
A disfunção da glicoproteína LAMP2 prejudica a fusão entre autofagossomos e lisossomos, resultando em degradação inadequada dos autofagossomos. Isso leva ao comprometimento cardíaco progressivo, caracterizado pelo acúmulo de vacúolos preenchidos com glicogênio. Imagens histológicas do miocárdio mostram a aparência vacuolizada típica dos cardiomiócitos (imagem A) e a substituição das áreas danificadas por fibrose (B). A cardiomiopatia hipertrófica pode ser identificada por meio de diversos exames diagnósticos. Os achados do eletrocardiograma tipicamente mostram sinais de hipertrofia cardíaca, frequentemente com aumento da voltagem do complexo QRS (D). O ecocardiograma comumente revela espessamento do septo e da parede posterior, como visto nas janelas paraesternal eixo curto (C) e apical quarto câmaras (E). Além disso, a ressonância magnética (RM) pode fornecer informações, como o realce tardio de gadolínio, visível nesta imagem de um estágio avançado de um fenótipo de cardiomiopatia dilatada (F). Por ser uma doença multissistêmica, a DD também pode se manifestar com atraso no desenvolvimento (AD) neurológico, miopatia e retinopatia.^6,12^

A análise histológica revela cardiomiócitos com vacuolização dramaticamente aumentada e características degenerativas.^21,22^ Além disso, a autofagia defeituosa resulta em morte celular e fibrose difusa, contribuindo para anormalidades de condução e maior suscetibilidade a arritmias.^21^ Acúmulo progressivo semelhante ocorre no músculo esquelético, levando à miopatia. Indivíduos com DD exibem variação leve a moderada no tamanho das miofibras e pequenos grânulos basofílicos dispersos, indicando o acúmulo de organelas lisossômicas nas miofibras.^21^

Achados neuropatológicos recentes sugerem que a *LAMP2* pode ter propriedades funcionais distintas em comparação com as do músculo. Alguns estudos propõem que a *LAMP2*A atua como um receptor para a autofagia mediada por chaperonas, que está implicada em distúrbios neurodegenerativos.^23^ Em neurônios, a deterioração gradual da acidificação lisossomal devido a danos oxidativos pode desencadear um ciclo vicioso, aumentando a produção de espécies reativas de oxigênio.^24,25^

A autofagia é crucial para proteger as células oculares contra danos oxidativos,^26^ mas a contribuição da *LAMP2* para a autofagia e a retinopatia permanece pouco compreendida.^27^ A expressão de *LAMP2*A e *LAMP2*B em linhagens celulares do epitélio pigmentar da retina (EPR) humano foi relatada, e a disfunção da *LAMP2* no EPR provavelmente causa patologia retiniana, incluindo distrofia de cones, por meio da formação prejudicada de autofagossomos.^6,27^

Recentemente, outros fatores modificadores da doença têm sido descritos, incluindo genes envolvidos na estrutura sarcomérica, no metabolismo energético e nas vias de degradação de proteínas, bem como fatores epigenéticos.^28,29^

### Diagnóstico

#### Critérios e abordagens diagnósticas para a doença de Danon

A DD apresenta uma gama de sintomas que variam de acordo com a idade e o sexo devido à sua herança ligada ao cromossomo X.^6^ Trata-se de uma condição progressiva e multissistêmica, embora as mulheres raramente apresentem envolvimento extracardíaco e frequentemente manifestem um fenótipo menos grave.^30^ As manifestações clínicas incluem CMH, miopatia esquelética e DI. O histórico familiar pode ser informativo, biópsias ósseas e miocárdicas podem ser úteis e o teste genético é necessário para um diagnóstico preciso.^6^

Um critério diagnóstico proposto para a DD envolve a identificação de uma PV ou LPV em *LAMP2* por meio de testes genéticos e a confirmação de pelo menos uma característica clínica da doença (para mulheres, apenas a apresentação cardiológica é considerada válida para o diagnóstico).^30^ As características clínicas utilizadas para o diagnóstico podem ser cardiológicas, musculares ou de neurodesenvolvimento, conforme descrito abaixo:

1. **Cardiológico:** Presença de uma alteração cardiovascular objetiva, como aumento da espessura do septo e da parede posterior, fração de ejeção reduzida (<50%) ou alterações no eletrocardiograma (fibrilação ou flutter atrial, taquicardia ou fibrilação ventricular, morte súbita cardíaca, alterações de repolarização, bloqueio atrioventricular ou padrões de pré-excitação devido à síndrome de Wolff-Parkinson-White ou vias fasciculoventriculares). Além disso, o realce tardio de gadolínio na RM cardíaca pode ser considerado.^6,30^
2. **Muscular:** Evidência de alterações musculares indicadas por eletromiografia anormal, avaliações funcionais (teste cardiopulmonar de exercício [CPET] ou teste de caminhada de 6 minutos [6MWT]), ou testes cronometrados E/OU níveis elevados de creatina quinase (CPK), AST (aspartato aminotransferase) ou ALT (alanina aminotransferase).^31,32^
3. **Neurodesenvolvimento:** Deficiência intelectual ou AD.^4,30^

### Manifestações clínicas e avaliações diagnósticas

#### Manifestações cardíacas

Em homens, geralmente se manifesta como hipertrofia concêntrica do ventrículo esquerdo com fração de ejeção normal. Em mulheres, a condição pode se manifestar de diversas formas, incluindo hipertrofia assimétrica, hipertrofia do ventrículo direito e fenótipos tanto hipertróficos quanto dilatados.^30^ A hipertrofia pode ser extrema, com espessamento acentuado do septo e da parede posterior, maior do que na maioria dos fenótipos ou genótipos semelhantes.

A autofagia disfuncional interrompe funções biológicas essenciais e, como o coração é um músculo constantemente ativo, essa interrupção leva a lesões miocárdicas contínuas, frequentemente evidenciadas pela elevação persistente da troponina.^11^ A doença tipicamente progride por meio da morte de cardiomiócitos e fibrose progressiva, resultando, em última instância, em insuficiência cardíaca com redução da fração de ejeção. Essa progressão pode levar ao afilamento e dilatação da parede. Permanece incerto se a cardiomiopatia dilatada em mulheres é uma continuação dessa progressão ou representa um fenótipo distinto.^21^ A gravidade da cardiomiopatia é um fator prognóstico significativo, e muitos pacientes podem necessitar de transplante cardíaco na segunda e terceira décadas de vida.^33^

O ecocardiograma é comumente usado para avaliar a função e as dimensões cardíacas, mas carece de especificidade para diferenciar os tipos de cardiomiopatia. A RM pode fornecer informações valiosas para a cardiomiopatia da DD.^30,34^ Em um estudo com 16 pacientes, 56% apresentaram um fenótipo de CMH simétrica, enquanto os demais apresentaram CMH assimétrica ou cardiomiopatia dilatada (apenas uma mulher neste estudo).^34^ Os achados da RM incluíram preservação do septo médio-basal (88%), envolvimento do ápice (100%), envolvimento da parede livre (94%), envolvimento subendocárdico (87%), alteração do sinal em T2 (44%) e alteração da perfusão em repouso (88%). Essas características sugerem considerar DD e a realização de testes genéticos para pacientes com realce tardio atípico (subendocárdico) em cardiopatia não-isquêmica, especialmente com preservação do septo médio-basal, envolvimento do ápice e envolvimento da parede livre.^34^

Arritmias atriais e ventriculares são prevalentes na DD, afetando quase todos os pacientes. O achado eletrocardiográfico mais comum é a pré-excitação com padrão de síndrome de Wolff-Parkinson-White (WPW), observada em 69% dos pacientes do sexo masculino e 27% do sexo feminino.^22^ Os padrões de WPW são observados nesses pacientes com uma frequência cinco vezes maior do que na CMH idiopática e familiar. Embora o mecanismo exato por trás das anomalias de pré-excitação não seja totalmente compreendido, pode estar relacionado à hipertrofia miocárdica ou à autofagia disfuncional.^35^ A alta voltagem no eletrocardiograma é frequentemente observada, sendo um dos sinais de hipertrofia cardíaca. Manifestações comuns incluem arritmias supraventriculares, como fibrilação atrial e flutter atrial, bem como arritmias ventriculares. Bloqueio atrioventricular e disfunção sinusal podem afetar até 50% dos pacientes e podem exigir implante de marca-passo. Em alguns casos, parada cardíaca ou morte súbita arrítmica podem ser o primeiro sinal da doença.^31^

#### Envolvimento do músculo esquelético

A miopatia na DD é significativamente mais comum em homens (80-90%) do que em mulheres (cerca de 5%).^31^ Geralmente se manifesta como fraqueza nos músculos proximais, incluindo os da cintura pélvica e os músculos axiais, e progride para envolver os ombros, o pescoço e as pernas. Apesar dessa fraqueza progressiva, raramente é incapacitante, e a maioria dos pacientes mantém a capacidade de andar até a idade adulta.

Um estudo clínico utilizando um dinamômetro para avaliar a força muscular mostrou que pacientes do sexo masculino com DD apresentam força significativamente reduzida em comparação com controles saudáveis. Níveis elevados de CPK sérica também podem ser observados.^32^

#### Aspectos neurológicos e psiquiátricos

Dificuldades de aprendizagem e déficits cognitivos podem estar presentes em 70 a 100% dos homens afetados e em 6 a 47% das mulheres, embora o comprometimento seja geralmente descrito como leve. Apesar desses desafios, a maioria dos indivíduos consegue viver de forma independente. Em uma revisão sistemática publicada em 2019, o comprometimento cognitivo foi observado em 47 dos 146 casos clínicos avaliados, representando 55,6% dos pacientes. A maioria dos indivíduos com comprometimento cognitivo era do sexo masculino (80%), em comparação com 10,3% do sexo feminino.^4^

Acidentes vasculares cerebrais embólicos podem ocorrer devido a trombos intracardíacos, especialmente no contexto de função ventricular esquerda deprimida, aneurismas apicais, fibrilação atrial ou flutter.^36^

Embora os transtornos de humor e ansiedade sejam mais prevalentes em pacientes com cardiomiopatia em comparação com a população em geral, os relatos específicos sobre a DD são limitados. Uma série de casos com 14 pacientes indicou que os transtornos psiquiátricos podem afetar até 69% dos indivíduos com DD,^37^ incluindo transtornos de humor (46,15% dos pacientes), transtornos de ansiedade (46,15%), transtorno de déficit de atenção/hiperatividade (TDAH) (15,4%) e, com menos frequência, transtorno desafiador opositivo e transtorno obsessivo-compulsivo (7,7% cada).^38^

### Manifestações gastrointestinais

A elevação das enzimas hepáticas é comum em pacientes com DD, particularmente em homens, com níveis médios de AST e ALT frequentemente superiores a três vezes o limite normal.^4,39^ A causa dessas elevações permanece incerta; possivelmente devido a lesão hepática pela ausência de *LAMP2* ou secundária ao aumento das pressões de enchimento do lado direito do coração. Além disso, níveis elevados de AST também podem refletir lesão muscular esquelética.^39^ Em casos avançados de insuficiência cardíaca, os pacientes também podem apresentar hepatopatia congestiva e hepatomegalia.

### Características oftalmológicas na doença de Danon: mecanismos e apresentação clínica

A deficiência visual é frequentemente relatada, sendo a retinopatia pigmentar e a miopia as alterações oculares mais comuns, embora a gravidade seja geralmente leve. Exames de imagem e testes funcionais revelam um fenótipo variável, com retinopatia pigmentar geralmente presente e potencialmente associada a depósitos retinianos e perda generalizada da função dos cones e bastonetes.^40^

O mecanismo por trás da retinopatia pigmentar está ligado ao acúmulo de remanescentes do segmento externo, mitocôndrias defeituosas e lipofuscina não degradável nas células do EPR.^41^ Estudos experimentais em camundongos knockout para *LAMP2* demonstram autofagia e fagocitose interrompidas nas células do EPR, levando ao acúmulo de lipídios e depósitos na lâmina basal, que resultam na perda de células do EPR e fotorreceptores.^42,43^

### Estratégias de teste genético e aconselhamento para a doença de Danon

A DD é herdada em um padrão dominante ligado ao cromossomo X.^34^ O diagnóstico é estabelecido em um homem com achados sugestivos pela identificação de uma PV) ou LPV hemizigótica em *LAMP2* por meio de teste genético, ou em uma mulher com uma PV ou LPV heterozigótica em *LAMP.*^27^ A identificação de uma variante hemizigótica ou heterozigótica de significado incerto em *LAMP2* não confirma nem descarta o diagnóstico.

O gene *LAMP2* apresenta aproximadamente 681 variantes germinativas descritas, incluindo 140 PV, 254 variantes de significado incerto (VUS) e 287 benignas. Entre as 140 PV, 89% (n=125/140) são de perda de função, 6 são mutações missense ou indels em fase e 9 são não codificantes. Entre as VUS, a grande maioria, 80% (n=204/254), são mutações missense ou indels em fase. Por fim, entre as variantes benignas descritas, 44% são não codificantes e 35,5% são sinônimas. Concluímos, em consonância com a literatura, que a perda de função é um mecanismo conhecido e descrito de doença na DI. Ao considerar as variantes relatadas em homens e mulheres, o mais importante a ter em mente é o fato de que os homens geralmente são mais gravemente afetados do que as mulheres, devido ao fato de possuírem apenas um cromossomo X, enquanto as mulheres possuem dois. Quando nos deparamos com mulheres sendo afetadas igualmente ou até mais gravemente do que os homens, isso deve levar a equipe médica a considerar (1) lionização, (2) mosaicismo somático ou mesmo (3) uma translocação entre o cromossomo X e um cromossomo autossômico.^6,28,44^

Finalmente, ao considerarmos uma doença de armazenamento lisossômico (DAL), falamos de perda de função em diferentes genes que levam à doença. É importante notar que, mesmo que a DD seja causada por variantes de perda de função, não há deficiência enzimática como em outras DALs, mas sim uma deficiência em uma glicoproteína transmembrana. Já em outras condições, especialmente as RASopatias, que frequentemente se apresentam com CMH e, às vezes, DI, falamos de variantes de ganho de função, sendo a neurofibromatose tipo 1 a exceção.^6,28,44^

A heterogeneidade clínica em pacientes do sexo feminino pode ser atribuída à extensão dos padrões de inativação do cromossomo X (XCI), que ocorrem em células femininas. Quando ocorre XCI aleatória, a sobreposição de domínios nucleares pode resgatar a expressão de *LAMP2* em fibras musculares esqueléticas, mas não em cardiomiócitos (que não se regeneram), explicando assim por que a maioria das pacientes do sexo feminino desenvolve cardiomiopatia, mas não miopatia esquelética. Um padrão de XCI aleatória foi encontrado em vários tecidos de diferentes pacientes.^4,43^

Se a mãe do probando tiver uma PV no gene *LAMP2*, a chance de transmiti-la em cada gravidez é de 50%. Homens com uma PV no gene *LAMP2* transmitirão a PV para todas as suas filhas e nenhum de seus filhos. Homens que herdarem a PV serão afetados; mulheres que herdarem a PV serão heterozigotas e poderão apresentar características de DI, geralmente não tão graves quanto as dos homens.

O teste genético deve ser realizado para diagnosticar o paciente e pode ser feito de forma direcionada a genes específicos, como testes de gene único ou painéis multigênicos, ou por meio de testes genômicos abrangentes, incluindo sequenciamento de exoma completo e sequenciamento de genoma completo. Enquanto o teste direcionado a genes exige que o médico determine quais genes provavelmente estão envolvidos, o teste genômico não exige isso.

Uma vez identificada a PV ou a LPV do gene *LAMP2* em um membro afetado da família, podem ser realizados testes pré-natais para gravidez de alto risco e testes genéticos pré-implantacionais. Essas possibilidades devem ser discutidas com o paciente e a família.^45,46^

Quando uma VUS é encontrada, devemos considerar que tal variante não constitui um diagnóstico de DD. As VUS devem ser reclassificadas de acordo com novos dados disponíveis na literatura, bancos de dados populacionais etc., à medida que o tempo passa. Às vezes, pode ser informativo segregar a variante e avaliar o fenótipo de outros membros da família possivelmente afetados para classificar ou reclassificar a variante adequadamente.

### Diagnóstico diferencial: Distinguindo a doença de Danon de outras doenças

Ao avaliar um paciente com suspeita de DD, outros diagnósticos devem ser considerados. Ao considerar condições genéticas, todos os sistemas devem ser avaliados, incluindo dismorfismos faciais, DI, hipotonia, ataxia e baixa estatura, entre outros. É importante verificar o histórico familiar, considerando etnia, outros membros da família e distribuição por sexo. Na Tabela 1, apresentamos uma lista dos principais diagnósticos diferenciais de DD.

**Tabela 1:** Condições genéticas a serem consideradas no diagnóstico diferencial da DD^4,30,31,47^

DOENÇA	VARIANTE GENÉTICA	DIFERENÇAS EM RELAÇÃO AO DD
CMH SARCOMÉRICA	*MYBPC3*, *MYH7*	PROGRESSÃO MAIS LENTA HIPERTROFIA ASSIMÉTRICA PRÉ-EXCITAÇÃO VENTRICULAR (RARA) PENETRÂNCIA INCOMPLETA COM FENÓTIPOS DIFERENTES DENTRO DA MESMA FAMÍLIA
SÍNDROME *PRKAG2*	*PRKAG2*	PRÉ-EXCITAÇÃO VENTRICULAR COM OU SEM CMH BLOQUEIOS ATRIOVENTRICULARES (COMUNS) TAQUICARDIA SUPRAVENTRICULAR A FIBROSE GERALMENTE ESTÁ AUSENTE.
ATAXIA DE FRIEDREICH	EXPANSÃO DE TRINUCLEOTÍDEOS *GAA* EM FXN	ATAXIA DA MARCHA COM DISARTRIA E DISFAGIA. MOVIMENTOS OCULARES ANORMAIS E ATROFIA ÓPTICA HIPERTROFIA VENTRICULAR ESQUERDA MENOS GRAVE INÍCIO DOS SINTOMAS POR VOLTA DOS 10-15 ANOS.
DOENÇA DE POMPE	*GAA*	INÍCIO NA INFÂNCIA COM ENVOLVIMENTO CARDÍACO E EXTRACARDÍACO GRAVE MACROGLOSSIA, DIFICULDADE PARA DEGLUTIÇÃO, BAIXO GANHO PONDEROESTATURAL ATRASO MOTOR / HIPOTONIA HEPATOMEGALIA NA INFÂNCIA
DOENÇAS MITOCONDRIAIS	VARIAÇÕES NO mDNA OU NO DNA NUCLEAR	FRAQUEZA MUSCULAR, PERDA AUDITIVA, CONVULSÕES, ATRASO NO DESENVOLVIMENTO, DEFICIÊNCIA VISUAL. PODE HAVER ENVOLVIMENTO DE MÚLTIPLOS ÓRGÃOS E SISTEMAS OS SINTOMAS PODEM PIORAR COM INFECÇÕES
RASOPATIAS	*NF1*, *NRAS*, *KRAS*, *PTPN11*, *SOS1*, *RAF1* E OUTRAS	DOENÇA CARDÍACA CONGÊNITA HIPERTELORISMO, PESCOÇO CURTO E MANIFESTAÇÕES CUTÂNEAS (MANCHAS CAFÉ-AU-LAIT, LENTIGINAS) BAIXA ESTATURA
DOENÇA DE FABRY	*GLA*	CARDIOMIOPATIA DE INÍCIO NA IDADE ADULTA CÓRNEA VERTICILATA, DEFICIÊNCIA VISUAL DOENÇA RENAL CRÔNICA ANGIOQUERATOMAS ACROPARESTESIA REDUÇÃO DA SUDORESE
MIOPATIA LIGADA AO X COM AUTOFAGIA EXCESSIVA	*VMA21*	CARDIOMIOPATIA HIPERTRÓFICA LEVE EM UMA MINORIA DE CASOS HIPOTONIA E ATROFIA MUSCULAR VACÚOLOS AUTOFÁGICOS EM BIÓPSIA MUSCULAR

### Estratégias atuais de manejo e considerações prognósticas na doença de Danon

A DD não está bem representada em coortes ou ensaios históricos de CMH, o que significa que as diretrizes existentes para CMH podem não abordar completamente seus aspectos únicos. Atualmente, não existem diretrizes de prática clínica específicas para DD, e o manejo depende muito da opinião de especialistas e da extrapolação de diretrizes gerais para cardiomiopatia.^47^

Um recente documento de posicionamento da Sociedade Italiana de Cardiologia (SIC) e da Sociedade Italiana de Cardiologia Pediátrica (SICP) sugere a redução do limiar para o implante de cardioversor-desfibrilador implantável (CDI) em pacientes com disfunção diastólica. Os CDIs devem ser considerados para pacientes com hipertrofia ventricular esquerda grave, taquicardia ventricular não sustentada ou síncope inexplicada.

Devido à sua natureza multissistêmica, a DD deve ser tratada em centros de referência especializados por uma equipe multidisciplinar.^33,47^

**Figura 1:**
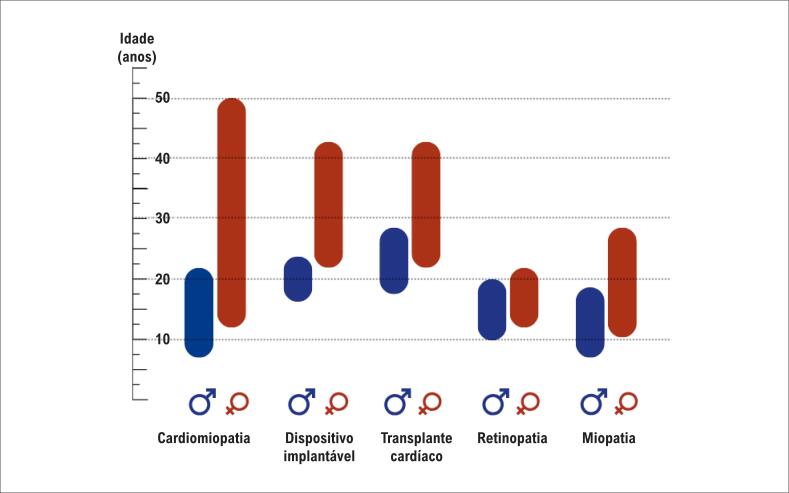
Evolução temporal das manifestações clínicas da doença de Danon de acordo com o sexo.^4,22,30,39,50^

Os indivíduos do sexo masculino geralmente apresentam sintomas cerca de 15 anos antes das mulheres (Figura 1), com diagnóstico frequentemente na infância ou adolescência, manifestando-se com CMH grave que progride para insuficiência cardíaca terminal na segunda ou terceira década de vida, com progressão para insuficiência cardíaca terminal ocorrendo uma década ou mais tarde do que nos homens.^4^ Além disso, as mulheres tendem a apresentar menor incidência de manifestações extracardíacas.^4,48–50^ O fenótipo em mulheres é mais variável, com penetrância e expressividade heterogêneas, provavelmente devido à inativação do cromossomo X enviesada e ao mosaicismo funcional da expressão de *LAMP2*. Embora raro, algumas mulheres exibem um fenótipo fulminante, provavelmente resultante da inativação do cromossomo X enviesada favorecendo a variante PV de *LAMP2.*^50^

Os CDI são geralmente indicados para homens na segunda década de vida e mulheres na terceira (Figura 1). À medida que a doença progride, o tratamento farmacológico da insuficiência cardíaca pode não resultar em melhora clínica, evoluindo para indicação de transplante, geralmente na segunda ou terceira década de vida.^6^ Em uma coorte de 38 pacientes com DD submetidos a transplante, a mediana de idade no momento do transplante foi de 20,2 anos. Apesar da progressão mais lenta da doença em mulheres, não foi encontrada diferença significativa de idade entre os sexos. A taxa de sobrevida em 5 anos após o transplante foi de 87,1%, semelhante à de outras cardiomiopatias.^33^

Embora não existam recomendações específicas para a imunossupressão pós-transplante, os inibidores de mTOR, como o sirolimus, representam um risco teórico de exacerbar a fraqueza muscular devido ao aumento da autofagia celular, embora esse efeito não tenha sido observado em dados recentes.^33^

Os resultados pós-transplante são geralmente favoráveis, apesar dos desafios decorrentes do envolvimento extracardíaco, como a doença muscular esquelética, que pode afetar a reabilitação. A DD não recorre no enxerto vascular do órgão, a miopatia esquelética progride muito lentamente após o transplante e a retinose pigmentar permanece a principal preocupação relacionada às manifestações sistêmicas da doença.^10,50^ Dada a raridade da DD, os estudos disponíveis são predominantemente observacionais e inerentemente propensos a viés de seleção, especialmente em relação aos resultados prognósticos.

### Direções presentes e futuras na pesquisa e terapêutica da doença de Danon

Nosso objetivo é aumentar o reconhecimento precoce da DD para que a prevenção de possíveis complicações, como transplante cardíaco ou mesmo morte súbita, se torne viável, levando, em última análise, a resultados mais favoráveis para os pacientes. Também destacamos a importância do aconselhamento genético, considerando o padrão de herança e como outros indivíduos de ambos os sexos podem ser afetados. O aconselhamento genético deve ser oferecido sempre que necessário.

Pesquisas futuras devem se concentrar no desenvolvimento de terapias direcionadas, como terapia gênica e tratamentos farmacológicos, com o objetivo de reduzir o acúmulo de autofagossomos ou melhorar a função da proteína *LAMP2*.

A terapia gênica tem o potencial de oferecer uma solução a longo prazo e até mesmo permanente para pacientes com DD, mas precisamos considerar os desafios, como o alto custo, a acessibilidade ao tratamento e a variabilidade na resposta entre os pacientes. O custo deve ser levado em conta, especialmente em países com muitas pessoas que dependem do sistema de saúde.

Um ensaio clínico inédito em humanos, ainda em andamento, demonstra que uma baixa dose da terapia gênica RP-A501 para DD foi geralmente bem tolerada, conferiu expressão do gene *LAMP2*B cardíaco e está associada a evidências preliminares de benefícios cardíacos e clínicos.^49^ O ensaio ainda apresenta limitações, como o pequeno número de indivíduos que receberam o tratamento e o foco apenas em homens com a doença.^50^

### Doença de Danon: Resumo do conhecimento atual e das necessidades futuras

Em resumo, a DD é uma doença rara que afeta principalmente o coração e os músculos esqueléticos, e geralmente apresenta um fenótipo mais grave em homens. O histórico familiar pode revelar indivíduos potencialmente afetados, e o teste genético é crucial.

Nosso principal objetivo é facilitar a disseminação do conhecimento sobre a DD para possibilitar o diagnóstico precoce e o apoio aos indivíduos afetados. Ainda há necessidade de pesquisas futuras sobre os mecanismos subjacentes das isoformas de *LAMP2* para aprimorar o leque de estratégias diagnósticas e terapêuticas.

## Data Availability

Os conteúdos subjacentes ao texto da pesquisa estão contidos no manuscrito.
