# Supplementary material for: Doença de Danon: Compreendendo o Papel das Variantes do LAMP2 na Miocardiopatia e no Envolvimento Multissistêmico
Source: Arq Bras Cardiol. 2026 Apr 17;123(3):e20250186. [Article in Portuguese] doi: 10.36660/abc.20250186 (PMC13128213; doi:10.36660/abc.20250186)
Supplement: Translation

# Translation: Danon Disease: Understanding the Role of *LAMP2* Variants in Cardiomyopathy and Multisystemic Involvement

**DOI:** 10.36660/abc.20250186i

**Authors:** Giovanna Napolitano Pereira Ribeiro, Layara Fernanda Vicente Pereira Lipari, Paula Mendonça Senra, Lucas Vieira Lacerda Pires, Marjorie Hayashida Mizuta, Natália Quintella Sangiorgi Olivetti, Vera Demarchi Aiello, José Eduardo Krieger

**Affiliations:** 1 São Paulo SP Brasil Laboratório de genética e cardiologia molecular - Instituto do Coração do Hospital das Clínicas da Faculdade de Medicina da Universidade de São Paulo, São Paulo, SP – Brasil

**Keywords:** Lysosomes, Hypertrophic Cardiomyopathy, Inborn Genetic Diseases

## Abstract

Danon disease (DD) is a rare X-linked disorder caused by pathogenic or likely pathogenic variants in the lysosome-associated membrane protein 2 (*LAMP2*) gene. It primarily manifests as hypertrophic cardiomyopathy, myopathy, intellectual disability, and retinopathy, with males typically experiencing a more severe and early-onset phenotype. The pathophysiology of DD is linked to defective autophagy due to LAMP2 deficiency, leading to the accumulation of glycogen-filled vacuoles within cardiomyocytes and other tissues. This results in progressive cardiac hypertrophy, conduction abnormalities, and eventual heart failure, often necessitating heart transplantation. Skeletal muscle involvement is common in males, while cognitive impairments are observed in both genders, though more prevalent in males. Diagnosis is challenging due to the condition's rarity and variable presentation, highlighting the need for a multidisciplinary approach, including genetic testing for definitive diagnosis. Genetic testing must be performed only after proper genetic counseling is done. Differential diagnosis must be considered when DD is suspected, depending on the signs and symptoms, such as Fabry disease, *PRKAG2*, Pompe disease, and others. Management strategies currently focus on symptomatic treatment, with cardiac transplantation as a critical intervention for advanced cases. Emerging therapies, such as gene therapy targeting *LAMP2*, could help in altering disease progression. This review provides a comprehensive overview of DD, discussing its clinical manifestations, underlying mechanisms, diagnostic challenges, and potential therapeutic approaches in improving outcomes. Our goal is to increase the recognition of DD so that the prevention of possible complications and early diagnosis becomes feasible, ultimately leading to more favorable patient outcomes.

## Introduction

Understanding Danon Disease: Genetics, Clinical Manifestations, and Pathophysiology

Danon disease (DD) is a dominant X-linked disorder caused by germline variants in the lysosome-associated membrane protein 2 (*LAMP2*) gene. It is clinically characterized by hypertrophic cardiomyopathy (HCM), myopathy, intellectual disability, and retinopathy.

The condition was first described by Danon et al. in 1981 in a report of two boys with cardiomyopathy, myopathy, and intellectual disability.^1^ In 2000, additional cases were reported, including 10 unrelated patients with vacuolar myopathy and HCM, all showing a deficiency in the LAMP2 protein and variants in the *LAMP2* gene.^2,3^

DD presents with a spectrum of symptoms, and due to its X-linked dominant inheritance, both hemizygous males and heterozygous females can be affected.^4^ Males typically develop progressive concentric left ventricular hypertrophy, often necessitating heart transplantation.^5^ Cardiac conduction abnormalities are also common. Skeletal muscle weakness may lead to delayed motor milestone acquisition, learning, or intellectual disabilities. In females, the disease tends to manifest later and progress more slowly, with more variable phenotypes.^4,6,7^

DD is challenging to diagnose, emphasizing the importance of a multidisciplinary approach for accurate diagnosis, including sensitive genetic counseling and tailored therapeutic strategies. As our understanding of DD pathophysiology deepens, there is hope for new treatments and improved outcomes. Sharing knowledge about rare conditions such as DD is essential to help clinicians suspect and properly diagnose potentially affected patients, thereby mitigating complications.

## Method

The authors conducted a systematic literature review following established scientific methodology to gather and analyze relevant studies in DD within the field of cardiogenetics. A comprehensive search was carried out across multiple academic databases, including PubMed, Embase, and Cochrane, using the keywords: "Danon disease"; "cardiomyopathy"; "LAMP2" and "lysosomal storage disorders". The selection criteria prioritized articles published in the last decade that directly addressed genetic and molecular aspects of the condition. The review focused particularly on articles that explored molecular mechanisms involved in the pathophysiology of DD.

During the review process, the authors also encountered a few conflicting findings among the selected studies, which were carefully analyzed and discussed among the authors in the context of methodological differences and variations in study populations, outcome definitions, and analytical approaches. All the figures and tables available in this article were developed by the authors, based on data extracted from previously published articles.

### Epidemiology

#### Global prevalence and diagnosis trends in Danon disease

The prevalence of DD remains unclear, but it is recognized to affect individuals across various countries and ethnicities^7^. Danon cardiomyopathy is progressive and typically characterized by a left hypertrophic ventricular phenotype. Among pediatric patients with HCM, DD has been identified as the underlying cause in 4% of cases.^5^ In certain subgroups, the prevalence is notably higher: up to 17% in individuals with both thickened left ventricular walls and pre-excitation^8^ and as high as 33% in male patients presenting with both vacuolar myopathy on muscle biopsy and HCM.^9^

According to a recent article, males tend to present with HCM 96,2% of the time, while females may present with either HCM (70.3%) or dilated cardiomyopathy (29.3%). But when it comes to heart transplantation, since the disease continues to progress, it occurs equally in both sexes.^4,10^

### Pathophysiology

#### *LAMP2* dysfunction in Danon disease: Mechanisms and cellular impact

The *LAMP2* gene, located on chromosome Xq24, encodes a fundamental lysosomal membrane glycoprotein crucial for lysosome biogenesis, lysosomal pH regulation, and autophagy.^11–16^ LAMP2 regulates lysosomal lumen pH, facilitating lysosomal acidification for optimal hydrolase activity.^16^ It also plays a significant role in chaperone-mediated autophagy, a process essential for the lysosomal degradation of proteins under stress and during normal protein turnover.^12–15^ The LAMP2 glycoprotein is also required for the proper fusion between autophagosomes and lysosomes during autophagy.^14^

A pathogenic (PV) or likely pathogenic (LPV) germline variant in *LAMP2* typically results in the loss or malfunction of the LAMP2 protein, leading to deficient autophagosome-lysosome fusion and impaired autophagosome degradation, leading to the accumulation of glycogen-filled vacuoles within cardiomyocytes and other tissues (as in the Central Illustration). There are three LAMP2 isoforms—LAMP2A, LAMP2B, and LAMP2C—which differ in their transmembrane and cytoplasmic domains but share identical luminal domains.^14,17–20^ Clinical features of DD are primarily mediated by the loss of the LAMP2B isoform, expressed in muscle tissue.^21^

**Figure:** Malfunction of the LAMP2 glycoprotein impairs the fusion between autophagosomes and lysosomes, resulting in inadequate degradation of autophagosomes. This leads to progressive cardiac involvement characterized by the accumulation of glycogen-filled vacuoles. Histological images of the myocardium show the typical vacuolated appearance of cardiomyocytes (image A) and the replacement of damaged areas by fibrosis (B). The HCM can be identified through various diagnostic exams. Electrocardiogram findings typically show signs of cardiac hypertrophy, often with increased QRS voltage (D). The echocardiogram commonly reveals thickening of the septum and posterior wall, as seen in the parasternal short-axis (C) and apical 4-chamber views (E). Additionally, magnetic resonance imaging can provide information, such as late gadolinium enhancement, which is visible in this image of a late-stage of a dilated cardiomyopathy phenotype (F). As a multisystemic disease, DD can also present with neurodevelopmental delay, myopathy and retinopathy. ^6,12^

Histological analysis reveals cardiomyocytes with dramatically increased vacuolation and degenerative features in cardiomyocites.^21,22^ Additionally, defective autophagy results in cell death and diffuse fibrosis, contributing to conduction abnormalities and increased susceptibility to arrhythmias.^21^ Similar progressive accumulation occurs in skeletal muscle, leading to myopathy. Individuals with DD exhibit mild to moderate variation in myofiber size and scattered small basophilic granules, indicating the accumulation of lysosomal organelles in myofibers.^21^

Recent neuropathological findings suggest that *LAMP2* may have distinct functional properties compared to those in muscle. Some studies propose that LAMP2A acts as a receptor for chaperone-mediated autophagy, which is implicated in neurodegenerative disorders.^23^ In neurons, gradual impairment of lysosomal acidification due to oxidative damage may trigger a vicious cycle, enhancing reactive oxygen species production.^24,25^

Autophagy is crucial for protecting eye cells against oxidative damage,^26^ but the contribution of *LAMP2* to autophagy and retinopathy remains poorly understood.^27^ The expression of LAMP2A and LAMP2B in human retinal pigment epithelium (RPE) cell lines has been reported, and LAMP2 dysfunction in RPE likely causes retinal pathology, including cone dystrophy, through impaired autophagosome formation.^6,27^

Recently, there have been other disease-modifying factors being described, including genes involved in sarcomeric structure, energy metabolism, and protein degradation pathways, as well as epigenetic factors.^28,29^

### Diagnosis

#### Diagnostic criteria and approaches for Danon disease

DD presents with a range of symptoms that vary by age and sex due to its X-linked inheritance.^6^ It is a progressive, multisystem condition, although women rarely exhibit extracardiac involvement and often present with a less severe phenotype.^30^ Clinical presentations include HCM, skeletal myopathy, and intellectual disability. Family history can be informative, skeletal and myocardial biopsies may be useful, and genetic testing is required for a precise diagnosis.^6^

A proposed diagnostic criterion for DD involves identifying a PV or LPV in *LAMP2* through genetic testing and confirming at least one clinical feature of the disease (for women, only the cardiological presentation is considered valid for diagnosis).^30^ The clinical features used for diagnosis can be cardiological, muscular, or neurodevelopmental, as outlined below:

1. **Cardiological:** Presence of an objective cardiovascular abnormality, such as increased septal and posterior wall thickness, reduced ejection fraction (<50%), or electrocardiogram abnormalities (atrial fibrillation or flutter, ventricular tachycardia or fibrillation, sudden cardiac death, repolarization abnormalities, atrioventricular block, or pre-excitation patterns due to Wolff-Parkinson-White syndrome or fasciculoventricular pathways). Additionally, late gadolinium enhancement on cardiac magnetic resonance imaging (MRI) may be considered.^6,30^
2. **Muscular:** Evidence of muscle abnormalities as indicated by an abnormal electromyography, functional assessments (cardiopulmonary exercise testing [CPET] or the 6-minute walk test [6MWT]), or timed tests AND/OR elevated levels of CPK, AST (aspartate aminotransferase) or ALT (alanine aminotransferase).^31,32^
3. **Neurodevelopmental:** Intellectual disability or developmental delay.^4,30^

### Clinical manifestations and diagnostic evaluations

#### Cardiac manifestations

In males, it generally appears as concentric left ventricular hypertrophy with a normal ejection fraction. In females, the condition can manifest in various forms, including asymmetric hypertrophy, right ventricular hypertrophy, and both hypertrophic and dilated phenotypes.^30^ The hypertrophy can be extreme, with a markedly thickened septum and posterior wall greater than most phenocopies or genocopies.

Defective autophagy disrupts essential biological functions, and since the heart is a constantly active muscle, this disruption leads to ongoing myocardial injury, often evidenced by persistent troponin elevation.^11^ The disease typically advances through cardiomyocyte death and progressive fibrosis, ultimately resulting in heart failure with reduced ejection fraction. This progression can lead to wall thinning and dilation. It remains uncertain whether dilated cardiomyopathy in females is a continuation of this progression or represents a distinct phenotype.^21^ The severity of cardiomyopathy is a significant prognostic factor, and many patients may require cardiac transplantation in their second and third decades.^33^

The echocardiogram is commonly used to assess cardiac function and dimensions, but lacks specificity for differentiating cardiomyopathy types. MRI can provide valuable information for DD cardiomyopathy.^30,34^ In a study of 16 patients, 56% showed a symmetric HCM phenotype, while others had asymmetric HCM or dilated cardiomyopathy (only one female in this study).^34^ MRI findings included mid-basal septum sparing (88%), apex involvement (100%), free wall involvement (94%), subendocardial involvement (87%), abnormal T2 signals (44%), and resting perfusion defects (88%). These features suggest considering DD and genetic testing for patients with atypical subendocardial late gadolinium enhancement in nonischemic cardiomyopathy, especially with mid-basal septum sparing, apex involvement, and free-wall involvement.^34^

Atrial and ventricular arrhythmias are prevalent in DD, affecting nearly all patients. The most common electrocardiographic finding is pre-excitation with a Wolf-Parkinson-White (WPW) syndrome pattern, seen in 69% of male and 27% of female patients.^22^ WPW patterns are observed in these patients at a rate five times higher than in idiopathic and familial HCM. While the exact mechanism behind pre-excitation anomalies is not fully understood, it may be related to myocardial hypertrophy or impaired autophagy.^35^ Elevated electrocardiogram voltage is frequently observed, indicative of cardiac hypertrophy. Common manifestations include supraventricular arrhythmias, such as atrial fibrillation and atrial flutter, as well as ventricular arrhythmias. Atrioventricular block and sinus dysfunction can affect up to 50% of patients and may require pacemaker implantation. In some cases, cardiac arrest or sudden arrhythmic death may be the first sign of the disease.^31^

### Skeletal muscle involvement

Myopathy in DD is significantly more common in males (80–90%) than in females (around 5%).^31^ It generally presents as weakness in proximal muscles, including those in the pelvic girdle and axial muscles, and progresses to involve the shoulders, neck, and legs. Despite this progressive weakness, it is rarely debilitating, and most patients retain the ability to walk into adulthood.

A clinical study using a dynamometer to assess muscle strength has shown that male patients with DD have significantly reduced strength compared to healthy controls. Elevated serum creatine kinase (CK) levels may also be observed.^32^

### Neurological and psychiatric aspects

Learning disabilities and cognitive deficits can be present in 70 to 100% of affected males and in 6 to 47% of females, though the impairment is usually described as mild. Despite these challenges, most individuals can live independently. In a systematic review published in 2019, cognitive impairment was observed in 47 of the 146 clinical cases evaluated, accounting for 55.6% of patients. The majority of those with cognitive impairment were male (80%), compared to 10.3% females.^4^

Embolic strokes can occur due to intracardiac thrombi, especially in the context of depressed left ventricular function, apical aneurysms, atrial fibrillation, or flutter.^36^

Although mood and anxiety disorders are more prevalent in cardiomyopathy patients compared to the general population, specific reports on DD are limited. A case series of 14 patients indicated that psychiatric disorders may affect up to 69% of individuals with DD,^37^ including mood disorders (46.15% of patients), anxiety disorders (46.15%), attention deficit/hyperactivity disorder (15.4%), and, less frequently, oppositional defiant disorder and obsessive-compulsive disorder (7.7% each).^38^

### Gastrointestinal manifestations

Elevations in liver enzymes are common among patients with DD, particularly in males, with mean levels of AST and ALT often exceeding three times the normal limit.^4,39^ The cause of these elevations remains uncertain; possibly due to hepatic injury from the absence of LAMP2 or secondary to increased right-sided filling pressures. Additionally, elevated AST levels could also reflect skeletal muscle injury.^39^ In advanced heart failure, patients may also experience congestive hepatopathy and hepatomegaly.

### Ophthalmologic features in Danon disease: Mechanisms and clinical presentation

Impaired vision is frequently reported, with pigmentary retinopathy and myopia being the most common ocular findings, though the severity is often mild. Clinical imaging and functional tests reveal a variable phenotype, with pigmentary retinopathy usually present and potentially associated with retinal deposits and generalized loss of cone and rod function.^40^

The mechanism behind pigmentary retinopathy is linked to the accumulation of outer segment remnants, defective mitochondria, and undegradable lipofuscin in RPE cells.^41^ Experimental studies in *LAMP2* knockout mice demonstrate disrupted autophagy and phagocytosis in RPE cells, leading to lipid accumulation and basal laminar deposits, which result in the loss of RPE and photoreceptor cells.^42,43^

### Genetic testing and counseling strategies for Danon disease

DD is inherited in an X-linked dominant pattern.^34^ The diagnosis is established in a male with suggestive findings by identifying a hemizygous PV or LPV in *LAMP2* through genetic testing or in a female with a heterozygous PV or LPV in *LAMP2*.^27^ Identification of a hemizygous or heterozygous *LAMP2* variant of uncertain significance does not establish or rule out the diagnosis.

The *LAMP2* gene has approximately 681 germline variants described, including 140 PV, 254 variants of uncertain significance (VUS), and 287 benign. Among the 140 PV, 89% (n=125/140) are loss of function, 6 missense or in-frame indels, and 9 non-coding. Among the VUS, the vast majority, 80% (n=204/254), are missense or in-frame indels. At last, among the benign variants described, 44% are non-coding, and 35,5% are synonymous. We conclude, what is according to the literature, that loss-of-function is a known and described mechanism of disease in DD. When considering the variants being reported in males and females, the most important thing to have in mind is the fact that males are usually more severely affected than females due to the fact that they only have one X chromosome, while women have two. When we come across women being equally or even more severely affected than males, it should lead the medical team to consider (1) lyonization, (2) somatic mosaicism, or even (3) a translocation between the X chromosome and an autosome chromosome.^6,28,44^

Finally, as we take into consideration a lysosomal storage disorder (LSD), we talk about loss-of-function in different genes leading to disease. It is important to note that even if DD is caused by loss of function variants, there is no enzymatic deficiency as in other LSD but a transmembrane glycoprotein. But in other conditions, especially RASopathies, that often present with HCM and sometimes intellectual disability, we talk about variants of gain-of-function, with neurofibromatosis type 1 being the exception.^6,28,44^

The clinical heterogeneity in female patients could be attributed to the extent of X-chromosome inactivation (XCI) patterns, which occur in female cells. When random XCI occurs, the overlap of nuclear domains can rescue *LAMP2* expression in skeletal muscle fibers but not in cardiomyocytes (which do not regenerate), thus explaining why most female patients develop cardiomyopathy but not skeletal myopathy. A random XCI pattern has been found in various tissues from different patients.^4,43^

If the mother of the proband has a *LAMP2* PV, the chance of transmitting it in each pregnancy is 50%. Males who have a PV in *LAMP2* will transmit the PV to all their daughters and none of their sons. Males who inherit the PV will be affected; females who inherit the PV will be heterozygous and may have features of DD, usually not as severe as those of the males.

Genetic testing must be performed to diagnose the patient, and it can be done in a gene-targeted manner, such as single-gene testing or multigene panel testing, or through comprehensive genomic testing, including whole exome sequencing and whole genome sequencing. While gene-targeted testing requires that the clinician determine which genes are likely involved, genomic testing does not.

Once the *LAMP2* PV or LPV has been identified in an affected family member, prenatal testing for a pregnancy at increased risk and preimplantation genetic testing can be performed. These possibilities must be discussed with the patient and family.^45,46^

When a VUS is found, we must consider that such a variant does not constitute a DD diagnosis. VUS must be reclassified according to new data available in literature, population databases, etc, as time passes. Sometimes, it is helpful to segregate the variant or other possibly affected family members to properly classify or reclassify the variant.

### Differential diagnosis: Distinguishing Danon disease from other conditions

When evaluating a patient for DD, other diagnoses should be considered. When considering genetic conditions, all systems must be assessed, evaluating any facial dysmorphisms, developmental delay, hypotonia, ataxia, and short stature, among others. It is important to check the family history, considering ethnicity, other family members, and gender distribution. In Table 1, we show a list of the main differential diagnoses of DD.

**Table 1:** Genetic conditions to consider in the differential diagnosis of DD^4,30,31,47^

DISEASE	GENETIC VARIANT	DIFFERENCES FROM DD
SARCOMERIC HCM	*MYBPC3*, *MYH7*	SLOWER PROGRESSION ASYMMETRIC PATTERN OF HYPERTROPHY VENTRICULAR PRE-EXCITATION (RARE) INCOMPLETE PENETRANCE WITH DIFFERENT PHENOTYPES WITHIN THE SAME FAMILY
PRKAG2 SYNDROME	*PRKAG2*	VENTRICULAR PREEXCITATION WITH OR WITHOUT HCM ATRIOVENTRICULAR BLOCKS (COMMON) SUPRAVENTRICULAR TACHYCARDIA FIBROSIS IS USUALLY ABSENT
FRIEDREICH ATAXIA	*GAA* TRINUCLEOTIDES EXPANSION IN *FXN*	GAIT ATAXIA WITH DYSARTHRIA, DYSPHAGIA ABNORMAL EYE MOVEMENTS AND OPTIC ATROPHY LESS SEVERE LEFT VENTRICULAR HYPERTROPHY INITIAL ONSET AROUND 10-15 YO
POMPE DISEASE	*GAA*	INFANT ONSET WITH SEVERE CARDIAC AND EXTRACARDIAC INVOLVEMENT MACROGLOSSIA, FEEDING DIFFICULTIES, FAILURE TO THRIVE MOTOR DELAY/HYPOTONIA HEPATOMEGALY IN INFANCY
MITOCHONDRIAL DISEASES	VARIANTS IN mDNA OR NUCLEAR DNA	MUSCLE WEAKNESS, HEARING LOSS, SEIZURES, DEVELOPMENTAL DELAY, VISUAL IMPAIRMENT SYMPTOMS EXACERBATE WITH INFECTION DIFFERENT ORGANS/SYSTEMS MAY BE INVOLVED
RASOPATHIES	*NF1*, *NRAS*, *KRAS*, *PTPN11*, *SOS1*, *RAF1* AND OTHERS	CONGENITAL HEART DISEASE HYPERTELORISM, SHORT NECK, AND CUTANEOUS MANIFESTATIONS (CAFÉ-AU-LAIT SPOTS, LENTIGINES) SHORT STATURE
FABRY DISEASE	*GLA*	ADULT-ONSET CARDIOMYOPATHY CORNEA VERTICILLATA, VISUAL IMPAIRMENT CHRONIC KIDNEY DISEASE ANGIOKERATOMAS ACROPARESTHESIA SWEATING ABNORMALITIES
X-LINKED MYOPATHY WITH EXCESSIVE AUTOPHAGY	*VMA21*	MILD HYPERTROPHIC CARDIOMYOPATHY IN A MINORITY OF CASES HYPOTONIA AND MUSCLE ATROPHY AUTOPHAGOCYTIC VACUOLES ON MUSCLE BIOPSY

### Current management strategies and prognostic considerations in Danon disease

DD is not well-represented in historical HCM cohorts or trials, meaning existing HCM guidelines may not fully address its unique aspects. Currently, there are no specific clinical practice guidelines for DD, and management relies heavily on expert opinion and extrapolation from general cardiomyopathy guidelines.^47^

A recent position paper by the Italian Society of Cardiology (SIC) and the Italian Society of Pediatric Cardiology (SICP) suggests lowering the threshold for implantable cardioverter-defibrillator (ICD) implantation in DD. ICDs should be considered for patients with severe left ventricular hypertrophy, non-sustained ventricular tachycardia, or unexplained syncope.

Given its multisystemic nature, DD should be managed at specialized referral centers by a multidisciplinary team.^33,47^

Male individuals typically experience symptoms about 15 years earlier than females, with diagnosis often in childhood or adolescence, presenting severe HCM that progresses to end-stage heart failure by the second or third life decade, with progression to end-stage heart failure occurring a decade or more later than in males.^4^ Additionally, females tend to have a lower incidence of extracardiac manifestations.^4,48–50^ The phenotype in females is more variable, with heterogeneous penetrance and expressivity likely due to skewed XCI and functional mosaicism of *LAMP2* expression. Although rare, some females exhibit a fulminant phenotype, likely resulting from skewed XCI favoring the *LAMP2* PV.^50^

ICD are typically indicated for males in their second decade and females in their third (Figure 1). As the disease progresses, heart failure treatment may become insufficient, requiring transplantation, typically by their second or third decade.^6^ In a cohort of 38 DD patients who underwent transplantation, the median age at transplant was 20.2 years (Figure 1). Despite slower disease progression in females, no significant age difference was found between sexes. The 5-year post-transplant survival rate was 87.1%, similar to other cardiomyopathies.^33^

**Figure 1:** Timeline evolution considering the clinical manifestations of Danon disease according to gender.^4,22,30,39,50^

While there are no specific recommendations for post-transplant immunosuppression, mTOR inhibitors like sirolimus pose a theoretical risk of exacerbating muscle weakness due to increased cellular autophagy, although this effect has not been observed in recent data.^33^

Post-transplant outcomes are generally favorable despite challenges from extracardiac involvement, such as skeletal muscle disease, which may affect rehabilitation. DD does not recur in the organ vascular graft, skeletal myopathy progresses very slowly after transplantation, and retinitis pigmentosa remains the major concern related to the systemic manifestations of the disease.^10,50^ Given the rarity of DD, available studies are predominantly observational and inherently prone to selection bias, especially in relation to prognostic outcomes.

### Present and future directions in Danon disease research and therapeutics

Our goal is to increase the early recognition of DD so that the prevention of possible complications such as cardiac transplantation or even sudden death becomes feasible, ultimately leading to more favorable patient outcomes. We also highlight the importance of genetic counseling, considering the mode of inheritance and how other individuals from both sexes may be affected. Genetic counseling should be offered as often as needed.

Future research should focus on developing targeted therapies, such as gene therapy and pharmacological treatments, aimed at reducing autophagosome accumulation or improving LAMP2 protein function.

Gene therapy has the potential to offer a long-term and even permanent solution to patients with DD, but we have to consider the challenges, such as the high cost, the treatment accessibility, and the response variability among patients. The cost must be considered, especially in countries with many individuals relying on the healthcare system.

An ongoing first-in-human trial demonstrates that a low dose of RP-A501 gene therapy for DD was generally well-tolerated, conferred cardiac *LAMP2B* gene expression, and is associated with preliminary evidence of cardiac and clinical benefits.^49^ The trial still has limitations with the small number of individuals receiving the treatment and the focus only on males with the condition.^50^

### Danon disease: Summarizing current knowledge and future needs

In summary, DD is a rare disorder that primarily affects the heart and skeletal muscles, and usually presents a more severe phenotype in males. Family history can reveal potentially affected individuals, and genetic testing is crucial.

Our primary aim is to facilitate the dissemination of knowledge about DD to enable early diagnosis and support affected individuals. There is still a need for future research concerning the investigation of the underlying mechanisms of *LAMP2* isoforms to improve the array of diagnostic and therapeutic strategies.

## Data Availability

The underlying content of the research text is contained within the manuscript.
